# Sex-dependent associations between maternal prenatal cortisol and child callous-unemotional traits: Findings from the Wirral Child Health and Development Study

**DOI:** 10.1016/j.psyneuen.2019.104409

**Published:** 2019-11

**Authors:** Nicola Wright, Andrew Pickles, Elizabeth C. Braithwaite, Helen Sharp, Jonathan Hill

**Affiliations:** a Institute of Psychiatry, Psychology and Neuroscience, Kings College London, London, UK; b Department of Psychology, Manchester Metropolitan University, Manchester, M15 6GX, UK; c Department of Psychological Sciences, Institute of Life and Human Sciences, Liverpool, UK; d School of Psychology and Clinical Language Sciences, University of Reading, Reading, UK

**Keywords:** Cortisol, Pregnancy, Callous-unemotional traits, Anxious-depressed symptoms, sex differences

## Abstract

**Background:**

Elevated maternal glucocorticoids during pregnancy may impact on fetal development in a sex-dependent way, leading to increased amygdala activation and increased risk for internalising disorders in females. Based on evidence implicating reduced amygdala activation in callous-unemotional (CU) traits, we predicted that elevated maternal cortisol in pregnancy would be associated with lower CU traits and elevated anxious-depressed symptoms, only in girls.

**Methods:**

Participants were 225 members of a stratified subsample within an epidemiological longitudinal cohort (WCHADS). Salivary cortisol was measured over two days at 32 weeks gestation (on waking, 30-min post-waking and during the evening) and the log of the area under the curve (LogAUC) was calculated as an index of diurnal cortisol. Mothers reported on child CU traits and anxious-depressed symptoms at 2.5, 3.5 and 5.0 years of age.

**Results:**

As predicted there was a sex of child by cortisol interaction (p < .001) whereby elevated maternal cortisol was associated with lower child CU traits, explaining 25% of the variance, in girls, but not in boys. This effect remained when controlling for relevant confounders and anxious-depressed symptoms. By contrast, elevated maternal cortisol did not predict higher anxious-depressed symptoms in girls.

**Conclusions:**

The study adds to growing evidence for sex-dependent effects of elevated maternal cortisol during pregnancy on early child psychopathology, consistent with mediation by elevated amygdala activation. The conditions under which, in girls, this is associated with heightened responsiveness to others’ distress characteristic of low CU traits, or with increased affective symptoms, require further study.

## Highlights

• Prenatal maternal cortisol predicts child behaviours up to age 5 years.
• The association shows marked sex differences.
• Higher cortisol is associated with lower callous-unemotional traits only in girls.
• Higher cortisol is not associated with child anxious-depressed symptoms.

## 1 Introduction

Conduct problems that become evident in early childhood confer a markedly elevated risk for later antisocial behaviours and personality disorder and a wide range of psychiatric and interpersonal difficulties (Hill, 2011). Understanding early mechanisms is therefore critical. The study of mechanisms in conduct disorders has been greatly enhanced by the identification of a distinctive pattern of social behaviours commonly referred to as ‘callous unemotional (CU) traits’ that appear to be associated with particularly persistent, severe and violent behaviours (Frick et al., 2014). In order to understand their role in early-appearing conduct problems, we need to study possible risk factors for the early development of CU traits. In spite of continuing uncertainties about how early CU traits become evident and can be measured, several studies have provided indications of very early vulnerabilities. Studies of infants have implicated lower face preference and less eye gaze with mothers, and also lower maternal sensitivity and positivity in the development of CU traits up to age 6.0 years (Bedford et al., 2015, 2017; Wright et al., 2018). These findings are consistent with evidence from studies of older children implicating both impaired eye contact and lower positive parenting (Dadds et al., 2012, 2014; Hawes et al., 2011). Also consistent with studies of older children, Waller et al. (2016) showed in an adoption study that fearlessness in biological mothers predicted child CU behaviours at age 27 months, mediated via infant fearlessness. The association was moderated by adoptive mothers’ positive parenting. In older children, CU traits are associated with lack of responsiveness to sad and fearful faces, and with fearlessness (Dadds et al., 2006, 2011; Frick et al., 2003; Leist and Dadds, 2009; Muñoz, 2009; Waller et al., 2016) both of which are known to be associated with reduced amygdala activation (Fusar-Poli et al., 2009; Murphy et al., 2003). Imaging studies with children with conduct problems and CU traits have shown that children with high CU traits have reduced connectivity between the amygdala and prefrontal cortex (Finger et al., 2012; Marsh et al., 2008), and that adults with psychopathic traits and aggression from childhood have lower amygdala volume (Pardini et al., 2014). Further, children with high levels of CU traits demonstrate lower right amygdala activity in response to viewing fearful faces (Jones et al., 2009; Marsh et al., 2008) and participating in theory of mind tasks (Sebastian et al., 2012). There is also evidence that reduced amygdala responses to a face-emotion processing task mediates the relationship between CU traits and proactive aggression (Lozier et al., 2014). Thus, this evidence suggests a key role of amygdala structure, connectivity and function in CU traits.

Structural and functional MRI evidence has shown that higher prenatal cortisol levels are associated with amygdala hyper-reactivity and with increased amygdala volumes, in a sex-dependent manner. Increased maternal cortisol in pregnancy has been associated with increased amygdala function (Graham et al., 2019) and volume (Buss et al., 2012) in girls only. In these studies, high prenatal cortisol was also positively associated with affective problems in girls, an effect which was, in part, mediated by right amygdala volume (Buss et al., 2012) and increased amygdala connectivity (Graham et al., 2019) during infancy. These findings are consistent with a large body of animal and human evidence showing a sex-dependent association between prenatal stress and HPA axis function, and post-natal HPA axis regulation and anxious and depressed behaviours in offspring. For example, in studies of the offspring of stress-exposed pregnant rats, females but not males show anxiety and depression-like behaviours, such as reduced exploration of the open arms of an elevated maze test (Zagron and Weinstock, 2006) and increased length of immobility in the forced swim test (Frye and Warwrzycki, 2003).

In the human literature, maternal prenatal cortisol has been linked to the following outcomes in females, but not males: reduced fetal growth, an established risk factor for later depression (Braithwaite et al., 2018); increased infant negative emotionality (Braithwaite et al., 2017a; Braithwaite et al., 2017b); fearful infant temperament (Sandman et al., 2013); affective problems in childhood (Buss et al., 2012); pre-adolescent anxiety (Sandman et al., 2013), depression and a flattened diurnal cortisol profile in adolescence (Van den Bergh et al., 2008). A similar sex-dependent association between prenatal maternal cortisol and newborn telomere length has also been reported recently (Enlow et al., 2019). Studies of self-reported maternal prenatal stress have also shown sex-specific associations, for example, in a publication from the Generation R cohort (n = 2546) mothers’ prenatal stress assessed as life events and long-standing difficulties predicted increased hair cortisol levels at age 5 years in girls but not boys (Molenaar et al., 2019). In a study with both self-reported and objective stress measurement, following mothers pregnant during the Iowa floods (n = 94), maternal prenatal stress was associated with increased cortisol reactivity in girls but not boys at age 2.5 years (Ping et al., 2015). Putting all these findings together, we hypothesised that elevated prenatal cortisol will be associated with decreased CU traits, and only in girls. In the light of the reported associations between maternal cortisol and child anxious and depressed symptoms, we also predicted that elevated maternal cortisol would be associated with higher levels of anxious-depressed symptoms, and only in girls.

## 2 Methods and materials

### 2.1 Design

Participants were members of the Wirral Child Health and Development Study (WCHADS), a prospective epidemiological study starting in pregnancy (see Sharp et al., 2012 for a detailed description of the sample). All women gave written informed consent at the point of recruitment in the antenatal clinic and at each subsequent assessment phase. The authors assert that all procedures contributing to this work comply with the ethical standards of the relevant national and institutional committees on human experimentation and with the Helsinki Declaration of 1975, as revised in 2008. All procedures involving human subjects/patients were approved by the Cheshire North and West Research Ethics committee. The study used a two stage stratified design in which a consecutive general population sample (the ‘extensive’ sample) is used to generate a smaller ‘intensive’ sample stratified by psychosocial risk with more detailed measurement over time, and both are followed in tandem. The sample stratification has been described previously (Sharp et al., 2012; Wright et al., 2019) but in brief, the stratification was based on maternal responses to questions about psychological abuse in their current or recent partner relationship (Moffit et al., 1997). The stratification variable was chosen for its known association with a variety of risk factors for early child development.

### 2.2 Sample

The whole cohort (extensive sample) comprised 1,233 women recruited in pregnancy with a live, singleton baby for long-term follow up post-birth. The mean age at recruitment was 26.8 years (SD = 5.8, range 18–51), 41.8% of the sample were in the most deprived quintile of UK neighbourhoods (Noble et al., 2004) and 96.1% were White British. This report uses the stratified subsample of mothers recruited to the intensive sample at 32 weeks pregnancy and available at birth for longitudinal follow-up (n = 316). Diurnal saliva samples were collected at 32 weeks gestation and were available for 239 of this sub-sample of mothers. Complete and usable cortisol data was provided by 225 mothers who form the sample used in this analysis; their demographic characteristics are presented in Table 1. Mothers reported on child behaviours and CU traits and their own depression symptoms when the children were aged 2.5 (mean age: 30.98 months, SD = 2.34), 3.5 (mean age: 41.78 months, SD = 1.96) and 5.0 years (mean age: 57.82, SD = 2.94). Mothers also reported on their own CU traits at child age 5.0 years. All data analyses were weighted to give estimated means and coefficients for the whole cohort of 1233.

**Table 1:** Demographic characteristics of the sample and descriptive statistics for key study variables by sex.

Variable	Female			Male		
	N	mean	SD	N	mean	SD
Maternal cortisol log mean AUC	120	1.12	0.39	105	1.15	0.45
Age 2.5 years CBCL Anxious-depressed total score	104	1.30	1.60	91	1.34	1.71
Age 3.5 years CBCL Anxious-depressed total score	104	1.43	1.50	93	1.48	1.52
Age 5 years CBCL Anxious-depressed total score	102	1.79	1.86	92	2.01	2.16
Age 2.5 years CU factor score	103	−0.07	0.46	91	0 .07	0.50
Age 3.5 years CU factor score	104	0.01	0.75	94	0.07	0.75
Age 5 years CU factor score	109	0.05	0.80	95	0.01	0.91
Sample stratification mid- stratum	120	5.8%		105	5.3%	
Sample stratification high-stratum	120	11.9%		105	12.9%	
Mother mid-range age at pregnancy	120	56.6%		105	60.0%	
Mother older age at pregnancy	120	27.1%		105	26.5%	
Mother education >18 years	120	58.0%		105	57.7%	
Mother smoked in pregnancy	120	15.0%		105	21.2%	
Living with partner at conception	120	79.1%		105	75.8%	
Most deprived quintile	120	44.3%		105	39.5%	
Mother DSM depression during pregnancy	120	14.7%		104	14.1%	
Mother anxiety symptoms 32 weeks pregnancy	120	31.58	8.44	105	32.15	9.20
Mother depression factor score from child age 2.5 years to 5 years	120	0.08	1.01	105	−0.10	0.99

*Note*: Mother mid-range age = 21–30 years, mother older age = >30 years.

### 2.3 Measures

#### 2.3.1 Maternal cortisol

At 32 weeks gestation, mothers collected saliva samples at home over two consecutive working days. Saliva was collected on waking, 30 min post-waking, and during the evening (approx. 12 h after waking (mean = 12 h 10 min, *SD* = 1 h 15 min)). Participants stored the samples in their freezer until a research assistant collected them 1–2 weeks later. Samples were stored at −20 °C before transportation to Imperial College London on dry ice for analysis. All samples were assayed for salivary cortisol using a commercially available immunoassay (Salimetrics, UK). Inter- and intra-assay variation were 7.9% and 8.9% respectively. Salivary assays were run in duplicate except for a small minority of cases with minimal volume (n = 3). In this study we used the log of the area under the curve (LogAUC) as an index of diurnal cortisol release. The area under the curve was calculated using the trapezoid method with respect to ground, taking the mean (over 2 days) of the awakening, 30 min and 12 h post-awakening measures. A full set of maternal saliva samples over two days was available for 230 participants. Of these, five cases were unusable due to time of collection not being recorded or researcher labelling error, resulting in a sample of 225. Ten participants provided saliva samples over one day, and their raw scores were used in analyses instead of a mean score. Cortisol measured across the two days was correlated at the three time-points (waking cortisol r = 0.485, p < .001, 30-min post-waking r = 0.473, p < .001, 12-h post-waking r = 0.157, p = .02).

#### 2.3.2 Child callous-unemotional (CU) traits

CU traits were assessed by mother-report at 2.5, 3.5 and 5.0 years. There is evidence that CU traits can be validly measured in preschool aged children, with a recent meta-analysis of 10 studies of children aged <5.0 years demonstrating that CU traits predict later problem behaviour over and above early problem behaviour (Longman et al., 2016). In this sample, CU traits were assessed using a combination of the Antisocial Processes Screening Device – Preschool Version (APSD; (Frick and Hare, 2001)) and items from the Child Behaviour Checklist (CBCL; (Achenbach and Rescorla, 2000)), the Brief Infant Toddler Assessment (BITSEA; (Briggs-Gowan et al., 2004)) and the Strengths and Difficulties Questionnaire (SDQ; (Goodman, 1997)). We have previously created CU traits latent factor scores at 2.5, 3.5 and 5.0 years (Wright et al., 2018) by subjecting items to exploratory and confirmatory factor analyses in MPlus (Muthén and Muthén, 2012). The items and their factor loadings are displayed in Table A1 (Appendix A in supplementary). The items at each age were allowed to vary to reflect developmental differences in the manifestation of CU traits and measurement invariance by sex was established for the measure at each age point. We have provided evidence for the validity of the age 5.0 CU traits measure in Wright et al. (2019) by demonstrating incremental prediction to physical aggression at age 7.0, controlling for age 5.0 aggression. For this analysis, a latent variable was created from the factor scores from the three age time points to represent CU traits from 2.5 to 5.0 years. The summary statistics for this variable, and the associations between the three age points have been published previously (Wright et al., 2018). We also report the results from a multi-group (by child sex) measurement model for the three CU time points in Appendix B. The model fit was excellent and the factor loadings were very similar across the three age points, and for boys and girls (2.5, 3.5 and 5.0 years were .69, .85 and .70 for boys and .70, .80 and .75 for girls).

#### 2.3.3 Child anxious-depressed symptoms

Maternal reports of child anxious-depressed symptoms were assessed at 2.5, 3.5 and 5.0 years using the CBCL (Achenbach and Rescorla, 2000). This measure has been used extensively in studies of child and adolescent emotional and behavioural disorders. The scale has 99 items, each scored 0 (not true), 1 (somewhat or sometimes true) and 2 (very true or often true), which are summed to create seven syndrome scales and two broadband internalising and externalising subscales. The total score on the 8 item anxious depressed syndrome scale was used in this analysis. A latent variable was created from the anxious-depressed symptom scale at each time point as a way of capturing long-term/persistent individual differences.

#### 2.3.4 Confounders

A number of potential confounding variables were selected because of their established association with child behavioural problems. This included mothers’ age at conception (0 => age 20 years, 1 = age 21 to 30 years, 2 => age 31 years), mothers’ age at leaving education (0 = age 18 or younger, 1 = age 19 years or older), marital status (0 = single or partner living elsewhere, 1 = married or cohabiting), smoking during pregnancy (0 = no smoking, 1 = smoking) and deprivation. Deprivation was assessed using the revised English Index of Multiple Deprivation (IMD); (Noble et al., 2004) based on data collected from the UK Census in 2001. According to this system, postcode areas in England are ranked from most deprived (i.e. IMD of 1) to least deprived (i.e. IMD of 32,482) based on deprivation in seven domains: income, employment, health, education and training, barriers to housing and services, living environment, and crime. All mothers were given IMD ranks according to the postcode of the area where they lived and assigned to a quintile based on the UK distribution of deprivation. A binary variable, with 1 = most deprived quintile versus 0 = all other quintiles, was used for analysis. Maternal depression, anxiety and CU traits were also included as possible confounds. Depression was assessed as any episode of DSM V major depression (American Psychological Association, 2000) during pregnancy. The semi-structured diagnostic interview the Schedule for Affective Disorders (SADS; Spitzer and Endicott, 1977) was administered at the 32 week gestation interview and at 7 months postpartum by trained graduate Research Assistants. For reliability purposes 28 audio recordings were independently rated and weighted kappa was acceptable, ranging from .83-1.00. Anxiety symptoms were assessed using the total score on the state scale of Speilberger State-Trait Anxiety Inventory, (STAI; (Spielberger et al., 1983)). Mothers’ CU traits in adult life were assessed at the age 5.0 assessment using the affective callousness subscale of the Self-Report of Psychopathy Scale (SRP (Paulhus et al., 2019 in press). The SRP was designed as a self-report measure of adult psychopathic features analogous to the Psychopathy Checklist-Revised (Hare, 2003).

Mothers’ depressive symptoms at the time of reporting child CU traits and anxious-depressed problems was included as a covariate to account for any potential bias on reporting. At ages 2.5 and 3.5 years maternal depressive symptoms were assessed using the Edinburgh Postnatal Depression Scale (EPDS; (Cox et al., 1987) and at age 5.0 years using the Centre for Epidemiological Studies - Depression (CES-D; (Radloff, 1977)). A single latent variable was created from the three time points for analysis.

### 2.4 Statistical analyses

**Fig. 1:**
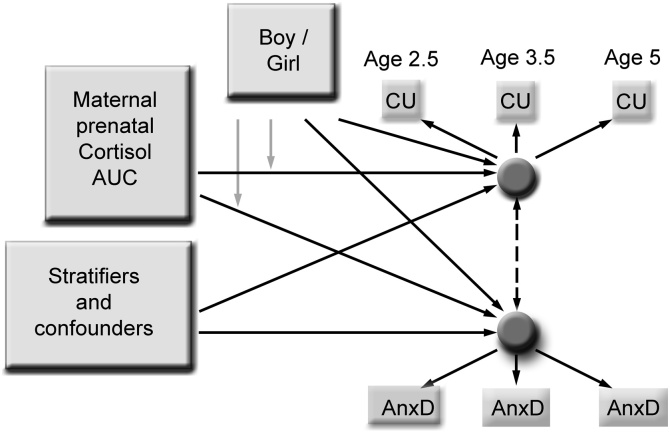
Schematic diagram of structural equation model using maternal prenatal cortisol, infant sex and covariates to predict the CU traits and anxious-depressed factors. Black lines refer to main effects and grey lines refer to interactions. Standardised estimates generated from this model are displayed in Table 2.

All analyses were undertaken in Stata version 14, primarily using the gsem command for structural equation models (SEM). The two-phase stratified sample design allows estimates to be reported for the general population by applying inverse probability weights. These weights were constructed to take account of the sample design stratification factor and additional variables associated with attrition and response on the variables required for the analysis: maternal age, depression, and smoking in pregnancy, years of education, marital status and the deprivation index for the mother’s home neighbourhood. The weights compensate for the systematic differences between our analysed sample and the whole population cohort. Modelled as latent variables in the SEMs (see Fig. 1) using a weighted maximum likelihood estimator, measures of child CU traits and anxious-depressed symptoms were not required to be complete. A single measure was deemed sufficient for inclusion in the model. The CBCL anxious-depressed total scores were highly skewed and so an ordinal variable was used in analyses. Test statistics and confidence intervals for coefficients were based on survey adjusted Wald tests (t-tests if single degrees of freedom (df) or F-tests if multiple df) using the robust ‘sandwich’ estimator of the parameter covariance matrix (Binder, 1983). All models included the paths corresponding to the main effects of sex, maternal prenatal cortisol and the interaction between maternal prenatal cortisol and sex shown in Fig. 1. In all models CU traits and anxious-depressed symptoms were modelled as simultaneous covarying outcome variables. Model 1 included no confounders; model 2 included confounders excluding the maternal postnatal depression and maternal CU traits scores; model 3 included confounders and maternal depression and CU traits. Two versions of Model 4 were estimated, Model 4a included an additional effect from the child anxious-depressed factor to the CU traits factor, and model 4b instead included the effect from the child CU traits to the anxious-depressed factor. These last two models were to clarify the specificity of effects, i.e. to test effects of maternal cortisol and sex on one factor while accounting for possible effects from the other. With an ordinal level outcome Stata gsem does not produce standardised output. To make coefficients for the same covariate more comparable across outcomes we report semi-standardised coefficients for outcomes standardised to unit variance by dividing unstandardized coefficients by the residual standard deviation of the outcome. Indicative R-squared values, being unavailable for weighted data, are reported from unweighted analyses. Continuous predictor variables were mean centred before inclusion in the models.

## 3 Results

### 3.1 Summary statistics

Descriptive statistics for all measures used in the analysis are displayed in Table 1. These showed very similar scores for boys and girls in the exposures and in the average behavioural outcomes. For ages 2.5 to 3.5 years, and 2.5 to 5.0 years and 3.5 to 5.0 years, the correlations among the CU traits factor scores were 0.63, 0.52 and 0.61 and for the CBCL anxious-depressed scores were 0.57, 0.50 and 0.46. The correlation between the CU and anxious-depressed latent variables created from the three age points was 0.32 (p < .001) in the total sample and 0.30 (p = .003) and 0.34 (p < .001) in boys and girls, respectively. The correlations between the key study variables on the total sample and in boys and girls separately are presented in Table C1 and C2 (Appendix C).

### 3.2 Main analysis

#### 3.2.1 CU traits

**Fig. 2:**
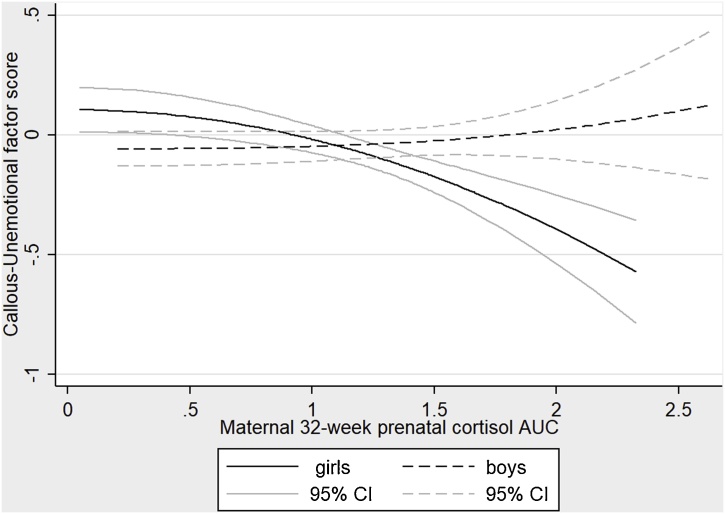
Sex-dependant associations between maternal prenatal cortisol and the CU traits factor score. The figure displays unweighted regression lines with 95% confidence intervals.

Table 2 displays the results of the structural equation models predicting child CU traits from maternal prenatal cortisol and child sex. In the simple model without confounders (Model 1) there were significant effects of child sex, maternal prenatal cortisol, and a sex by cortisol interaction on child CU traits. The main effect for cortisol corresponds to the estimated effect for girls, while that for boys is the sum of the effect for girls and the cortisol by gender interaction term. Testing these summed coefficients indicated that for boys, maternal cortisol was not associated with CU traits (p = .793, unweighted r^2^ = .01), while for girls, increased maternal cortisol was associated with lower CU traits (est = −0.476, p < .001, unweighted r^2^ = .25) (displayed in Fig. 2). The addition of confounders (sample stratifier, maternal age, maternal education, smoking in pregnancy, mother not living with partner, neighbourhood deprivation, prenatal maternal anxiety symptoms, and depression diagnoses) in Model 2 had little impact on the findings. In Model 3, maternal depression at the times of reporting the outcomes and mothers CU traits were added as confounds. Maternal depression and maternal CU traits significantly predicted higher child CU traits, but again the pattern and significance of the child sex and cortisol effects were unchanged. In view of the moderate correlation between CU traits and anxious-depressed symptoms, and in order to test whether the sex by cortisol interaction was specific to CU traits, in Model 4a a path was added from the anxious-depressed factor to the CU traits factor to examine the effect of maternal cortisol and sex on child CU traits after accounting for anxious-depressed symptoms. Although the anxious-depressed factor predicted CU traits, the estimated effects of cortisol and child sex, and their interaction, on CU traits retained the same pattern, somewhat reduced in magnitude but still clearly significant. The effect of maternal depression at the time of reporting on child CU traits was substantially reduced after the addition of this path, whereas mothers’ CU traits remained a substantial predictor of child CU traits.

**Table 2:** SEM estimates of covariate effects on the CU traits factor and on the anxious-depressed factor for a weighted sample of 225 children.

		Effects on CU traits		Effects on Anxiety-Depression score	
Model	Covariate	Coefficient	p	Coefficient	p
1	No confounders				
	Male sex	0.224	.179	0.041	.814
	Maternal cortisol	−1.586	<.001	−0.595	.047
	Sex X maternal cortisol	1.690	<.001	0.815	.060
2	Confounders (not shown)				
	Male sex	0.210	.174	0.028	.868
	Maternal cortisol	−1.238	<.001	−0.437	.168
	Male sex X maternal cortisol	1.469	<.001	0.479	.269
3	Confounders (not shown)				
	Male sex	0.331	.049	0.012	.949
	Maternal cortisol	−1.496	<.001	−0.545	.091
	Male sex X maternal cortisol	1.714	<.001	0.480	.301
	Maternal post-natal depression	0.094	.001	0.137	<.001
	Mothers CU traits	0.964	<.001	0.671	.015
4(a,b)	Confounders (not shown)				
	Male sex	0.390	.032	−0.078	.700
	Maternal cortisol	−1.229	<.001	−0.214	.574
	Male sex X maternal cortisol	1.338	.004	0.192	.716
	Maternal postnatal depression	0.046	.153	0.121	<.001
	Maternal CU traits	0.768	.005	0.539	.090
	Child Anxious D factor	0.212	.002		
	Child CU factor			1.257	<.001

#### 3.2.2 Anxious-depressed symptoms

The same set of models were then estimated predicting child anxious-depressed symptoms. Prior to the inclusion of confounds and child CU traits, there was an interaction between sex of child and maternal cortisol in the opposite from the predicted direction (Table 2, Model 1, est = 0.815, p = .060) with higher cortisol associated with lower anxious-depressed symptoms (est = 1.153, p = .091) in girls. This association was substantially reduced and became non-significant after the inclusion of confounds and current maternal depression and mothers’ CU traits (Models 2 and 3, Table 2). After the addition of child CU traits to examine for the specific association between maternal cortisol and child anxious-depressed symptoms, the direction of association was no longer opposite to our prediction, and entirely non-significant (Table 2, Model 4b). The estimated effect of mothers CU traits on child anxious-depressed symptoms became non-significant whilst the effect of maternal depression was unchanged.

## 4 Discussion

Based on recent evidence for sex-dependent associations between prenatal maternal cortisol and amygdala function (Graham et al., 2019), and known associations between amygdala under-activity and CU traits, as well as amygdala hyper-activity and internalising problems, we predicted that elevated prenatal maternal cortisol would be associated with reduced CU traits and increased anxious-depressed symptoms only in girls. Using data from a prospective, longitudinal cohort stratified by risk, we examined sex-specific associations between maternal prenatal cortisol at 32 weeks gestation and child CU traits assessed at three time points from ages 2.5 to 5.0 years. There was a negative association between maternal prenatal cortisol and CU traits, indicating that higher prenatal cortisol was associated with lower CU traits, moderated by sex of child. Higher maternal cortisol was associated with lower CU traits only in girls. This effect was evident when controlling for relevant confounding variables, and when controlling for child anxious-depressed symptoms. However, the hypothesis that increased maternal cortisol would be associated with increased anxious-depressed symptoms was not supported.

Our findings are consistent with an accumulating literature that supports the existence of sex-dependent glucocorticoid mechanisms for early child behaviours. Previously, the focus has been on the implications of increased negative emotionality (Braithwaite et al., 2017a; Braithwaite et al., 2017b, 2018), fearfulness (Sandman et al., 2013), anxiety and depression (Sandman et al., 2013; Van den Bergh et al., 2008), with indications of mediation by the amygdala (Buss et al., 2012; Graham et al., 2019) in girls, but not boys. Here we show, consistent with evidence that decreased amygdala activity is associated with elevated CU traits, that increased maternal cortisol in pregnancy confers protection against the development of CU traits in girls. The findings provide further evidence for female specific pathways linking prenatal cortisol exposure to altered brain structure and risk for child symptoms. The mechanisms underlying the effects of prenatal stress are not yet understood, but it seems likely that alterations in the function of the placenta contribute (O’Donnell et al., 2009; St-Pierre et al., 2016). The placenta modulates the passage of cortisol (O’Donnell et al., 2012) and other factors such as 5-HT (Bonnin and Levitt, 2012), to the fetus, and these may change neurodevelopment. In animal studies, prenatal stress has been shown to cause a down regulation of the cortisol metabolising enzyme 11β-hydroxysteroid dehydrogenase type II (11β-HSD2} (Mairesse et al., 2007) and we have evidence that the same also occurs in humans (Jensen et al., 2012; O’Donnell et al., 2012). Critically, female placentae are characterised by higher levels of 11 β-HSD2 and appear to be more responsive to the environment (Murphy et al., 2005). Studies have reported reduced 11 β-HSD2 levels in female, but not male, placentae exposed to prenatal maternal asthma (Murphy et al., 2005) and maternal prenatal stress (Mina et al., 2015). In fetuses born small for gestational age, female placantae show lower 11 β-HSD2 activity compared to male placentae (Mericq et al., 2009). Thus it appears that under adverse environmental conditions, female placentae adopt a strategy where they remain sensitive to maternal cortisol levels, whereas the male strategy is to become insensitive, instead opting for increased growth at the expense of lower survival (Clifton, 2010). There are indications also that epigenetic programming of the HPA axis contributes to sex-dependant effects of prenatal stress. Research has focused on NR3C1, the gene responsible for encoding the glucocorticoid receptor (GR). Higher DNA methylation of NR3C1 has been associated with prenatal depression and stress (Braithwaite et al., 2015; Hompes et al., 2013). NR3C1 methylation results in decreased GR expression which causes an overactive stress response in the offspring (Oberlander et al., 2008). Importantly, there is evidence that this effect is sex-specific, with research showing increased methylation for female infants (Ostlund et al., 2016; Stonawski et al., 2019) compared to males infants.

The present findings are consistent with the proposal that we have made previously, that the lower antisocial behaviour problems seen in girls compared to boys during childhood, and the increased anxious-depressed, and particularly depressive symptoms seen in girls after puberty, may be two sides of the same coin. In the context of childhood conduct problems, low CU traits are likely to be protective, but they also reflect heightened social sensitivity and prosocial behaviours that under some conditions may create risk for later depression. For example Murray et al. (2006) found that daughters, but not sons, of mothers with postnatal depression showed greater portrayal of prosocial behaviours in their doll play at age 5.0, which mediated the association between postnatal depression and adolescent depression (Hill et al., 2010).

Contrary to predictions, we did not find that prenatal cortisol was associated with increased anxious-depressed symptoms assessed from age 2.5 to 5.0 years. This was particularly surprising as we have evidence from the WCHADS of a sex dependent association between maternal cortisol and negative emotionality at age 9 weeks, with elevated cortisol associated with negative emotionality, only in girls (Braithwaite et al., 2017a; Braithwaite et al., 2017b). A review of studies of prenatal cortisol and child emotional and behavioural outcomes has highlighted that findings have been more consistent when examining outcomes in the first months of life than at later developmental stages (Zijlmans et al., 2015). There are also fewer studies later in development, and differences in samples, cortisol measurement and the use of different internalising problems outcomes make it difficult to draw firm conclusions. However, the inconsistency may also reflect developmental changes in the underpinning of emotional symptoms, suggesting further work is needed on the developmental pathway from early elevated emotionality and emerging social and emotional responsiveness.

Recent reviews have highlighted the need for more work on sex differences in the aetiology of CU traits (Viding and McRory, 2012) and the present findings add to this small and rather inconsistent literature. For example, Fontaine et al. (2010) found sex differences in MZ-DZ concordance for estimated trajectories of CU traits at age 7, 9 and 12 years. In boys there was a strong genetic effect, but in girls shared environment made the major contribution. By contrast, in a smaller twin sample of 10 year olds, a direct test of sex differences found no support for differences between boys and girls in genetic effects on CU traits. Similarly, a recent study reported an association between increased anterior insula volume and CU traits in boys only (Raschle et al., 2018), in contrast to previous studies, suggesting possible associations between decreased anterior insula function and volume and antisocial behaviour problems (Lockwood et al., 2013; Rogers and De Brito, 2016). Marked differences in sampling and measurement across the studies limits the scope to evaluate these inconsistencies.

In this study we found a moderate association between CU traits and anxious-depressed symptoms. This runs counter to early reports from clinical and community samples that conduct disordered children with high CU traits did not have elevated anxious-depressed symptoms (Hill, 2011). It also runs counter to theoretical accounts of adult psychopathy, which have argued that low anxiety is part of the psychopathy construct (Cleckley, 1976; Lykken, 1995). However, many of the studies of CU traits and anxiety in childhood which have reported zero or even negative associations between the two have done so after controlling for conduct problems (e.g. Frick et al., 1999; Pardini and Fite, 2010). In population studies examining simple correlations, small positive correlations have been found (Ezpeleta et al., 2013; Essau et al., 2006; Barker and Salekin, 2012). Associations found between CU traits and anxiety or internalising problems may be caused by measurement issues such as item overlap or effects of reporter bias. However they may also arise from a subgroup of children with the combination of elevated CU traits and anxiety symptoms, thought to be associated with exposure to early maltreatment, and referred to as secondary CU traits (Kimonis et al., 2012; Meehan et al., 2017). The importance of assessing both dimensions and of accounting for possible confounds and sources of reporter bias was underlined in our analyses. In a simple model, there was a sex by maternal cortisol interaction in the opposite of the predicted direction, which was no longer the case after the inclusion of relevant confounders and CU traits in the model.

### 4.1 Strengths and limitations

A significant strength of this study is the epidemiological sample recruited during pregnancy with a subsample stratified by psychosocial risk for more detailed assessment. This enabled data from the time consuming and costly measures of maternal prenatal cortisol to be weighted back to the general population. The measures were assessed prospectively and included relevant prenatal confounding variables, and measures of maternal mood that might have biased maternal reporting of CU traits and anxious-depressed symptoms. Essential for this study of sex differences, we demonstrated that the CU traits measures were invariant by sex. Further, these measures were created specifically to address the widely described issue of poor internal reliability of CU traits measures in young children. The latent variable approach to modelling the CU traits across three time points increased the robustness of the results by reducing the influence of measurement error. It also allowed us to examine a CU traits outcome that reflected persistence of CU traits, likely to be associated with poorer outcomes later in childhood. The loadings of age 2.5, 3.5 and 5.0 years CU traits scores were very similar, indicating that the earliest and latest measures contribute similarly to a preschool CU traits latent variable.

Limitations include that participants self-reported the timing of the cortisol sample collection, and we did not gather information on time spent asleep before the first morning sample, or information on the time of the last meal/drink before each cortisol sample, all of which could have introduced inaccuracies (Clow et al., 2004; Elder et al., 2014). The study did not include brain imaging so we could not examine whether the cortisol-CU traits association was mediated via variations in amygdala structure or function. CU traits and anxious-depressed symptoms were also assessed using solely maternal report, and the sample was almost exclusively White British so the findings may not be generalizable to other ethnic groups. We also cannot exclude potential common genetic influences on maternal HPA-axis functioning and children’s CU traits, however, we did include maternal depression and maternal CU traits as an attempt to address potential genetic confounds. Further as CU traits were assessed solely by mother report, we cannot rule out the possibility of a reporting bias, such that a mother’s cortisol levels during pregnancy may influence her interpretation of her daughter’s behaviour. Finally, whilst we find a large effect in the association between maternal cortisol and CU traits in girls, which we predicted from evidence on the role of amygdala under-activity, it is likely that the developmental pathways to CU traits are more complex. For example, there is evidence that higher physiological and emotional reactivity in infancy is associated with higher CU traits in middle childhood, when studies of older children and adults implicate lower physiological and emotional reactivity (e.g. Anastassiou-Hadjicharalambous and Warden, 2008; de Weid et al., 2012). In a large sample followed longitudinally to first grade, Wagner et al. (2019) found that the combination of high physiological arousal (assessed as resting cortisol) and harsh parenting in infancy predicted higher CU traits in first grade. Further, in the same sample, higher behavioural fear at 15 months predicted membership in a high CU traits/high ODD group in first grade (Mills-Koonce et al., 2015). However, in another study with measurement in infancy, lower negative reactivity to and greater recovery from the Still-Face procedure at age 7 months predicted membership in a high CU/high ODD group compared to ODD-only or no-ODD groups at age 3 years, although the group sizes in this study were very small (CU + ODD group n = 9; Willoughby et al., 2011). This is consistent with Waller et al.’s (2016) report of an association between lower observed fear in response to a novel scary toy at age 18 months and CU traits assessed at 27 months. It is possible that there are multiple distinct pathways to the development of child CU traits, one characterised by reduced emotional and physiological responsiveness and one characterised by elevated emotional and physiological responsiveness. These distinct pathways may represent the development of CU traits with and without anxiety. The present findings are consistent with there being a low emotional responsiveness pathway to CU traits implicating fetal adaptations. It remains to be established whether an elevated emotional and physiological responsiveness pathway also entail fetal and sex dependent mechanisms.

### 4.2 Future directions

We now have indications of a number of possible influences on the early development of CU traits, including low face preference (Bedford et al., 2015), low positive parenting (in particular, low sensitive responding, particularly to distress and low warmth; Wright et al., 2018), increased harsh parenting (Wagner et al., 2019), both higher (Mills-Koonce et al., 2015; Wagner et al., 2019) and lower (Willoughby et al., 2011; Waller et al., 2016) physiological and emotional reactivity, and now potentially female-specific prenatal glucocorticoid mediated influences. However, we do not yet understand whether these factors all contribute independently or in interaction and which are sex-dependant. Future work will need to examine the interplay between prenatal influences, infant social responsiveness and emotional availability, infant emotional and physiological reactivity, and parental warmth and sensitivity to emotions. We show a large effect in the association between maternal prenatal cortisol and later CU traits in girls, an important next step will be to examine potential mediators and moderators of this association, particularly during infancy. This should include parenting behaviours, the infants own HPA-axis functioning and very early markers of social responsiveness. The identification of sex-specific mechanisms in the development of CU traits is important given that most studies find lower rates of CU traits in females (e.g. Fontaine et al., 2010; Frick et al., 2003). It may be that the protective role of maternal cortisol in the development of CU traits in girls may contribute to the sex difference in rates of CU traits, and future work should seek to identify other processes which may contribute to the sex difference in rates.

### 4.3 Conclusion

We provide first evidence that increased maternal cortisol during pregnancy confers a protection against the development of CU traits in girls. The finding is consistent with existing evidence indicating that prenatal cortisol is associated with amygdala activity (Graham et al., 2019) and that amygdala activity is associated with elevated CU traits (e.g. Marsh et al., 2008). The findings are novel in identifying a very early and sex-dependant mechanism in the aetiology of childhood CU traits. More broadly, the findings are relevant to the emerging study of sex-dependent pathways to child and adolescence, and ultimately adult psychiatric disorders (Goldstein et al., 2014).

## CRediT authorship contribution statement

**Nicola Wright:** Conceptualization, Data curation, Formal analysis, Investigation, Methodology, Project administration, Visualization, Writing - original draft, Writing - review & editing. **Andrew Pickles:** Conceptualization, Data curation, Formal analysis, Funding acquisition, Investigation, Methodology, Project administration, Resources, Software, Supervision, Visualization, Writing - original draft, Writing - review & editing. **Elizabeth C. Braithwaite:** Conceptualization, Visualization, Writing - original draft, Writing - review & editing. **Helen Sharp:** Conceptualization, Data curation, Funding acquisition, Investigation, Methodology, Project administration, Resources, Supervision, Writing - review & editing. **Jonathan Hill:** Conceptualization, Data curation, Funding acquisition, Investigation, Methodology, Project administration, Resources, Supervision, Writing - original draft, Writing - review & editing.

## Declaration of Competing Interest

None
