# Supplementary material for: Sex-dependent associations between maternal prenatal cortisol and child callous-unemotional traits: Findings from the Wirral Child Health and Development Study
Source: Psychoneuroendocrinology. 2019 Nov;109:104409. doi: 10.1016/j.psyneuen.2019.104409 (PMC6857434; doi:10.1016/j.psyneuen.2019.104409)
Supplement: Supplementary file 1 [file mmc1.docx]

**APPENDIX A: CU traits items and their factor loadings (Wright et al. 2018)**

**Table A1.** Standardised CFA factor loadings for the CU traits items from Wright et al. (2018)

| **Items** | **Age 2.5** | **Age 3.5** | **Age 5** |
| --- | --- | --- | --- |
| **CU traits items** | | | |
| APSD 1: Concerned about the feelings of others (R) | .48 | .42 | .41 |
| APSD 2: Seems motivated to do his/her best in structured activities (R) | .61 | .37 |  |
| APSD 3: Is good at keeping promises (R) | .54 | .51 | .49 |
| APSD 4: Feels bad or guilty when he/she does something wrong (R) | .48 | .46 | .61 |
| APSD 5: Keeps the same friends (R) | .36 | .16 | .49 |
| APSD 6: Does not show emotions |  |  |  |
| CBCL 14. Cruel to animals | .93 |  | .59 |
| CBCL 58: Punishment doesn’t change his/her behavior | .62 | .74 | .68 |
| CBCL 67: Seems unresponsive to affection | .77 | .69 | .81 |
| CBCL 69: Selfish or won’t share | .42 |  |  |
| CBCL 70: Shows little affection toward people | .48 | .75 | .82 |
| CBCL 72: Shows too little fear of getting hurt |  | .49 |  |
| BITSEA 22. Tries to help if someone is hurt (R) | .69 |  |  |
| SDQ 1: Considerate of other people’s feelings (R) |  |  | .82 |
| SDQ 4: Shares readily with other children (R) |  |  | .60 |
| SDQ 9: Helpful if someone is hurt, upset or feelings ill (R) |  |  | .75 |
| SDQ 17: Kind to younger children (R) |  |  | .70 |
| SDQ 20: Often volunteers to help others (R) |  |  | .56 |

Note: *APSD = Antisocial Personality Screening Device, BITSEA = Brief Infant Toddler Social and Emotional Assessment, SDQ = Strengths and Difficulties Questionnaire*

**APPENDIX B: Multi-group measurement model for the three CU traits time points**

A multi-group by child sex measurement model was estimated on the age 2.5, 3.5 and 5 year CU traits factor scores. The standardised factor loadings for boys/girls are reported in Figure B2. The model showed excellent fit (chi2(4)=1.89, p=.755, RMSEA = .00 [90% CI .01-.10], CFI = 1.00, TLI = 1.00). There was a significant difference (p= .044) in the CU traits factor mean, with boys scoring higher than girls. All parameters in the model showed invariance by sex.


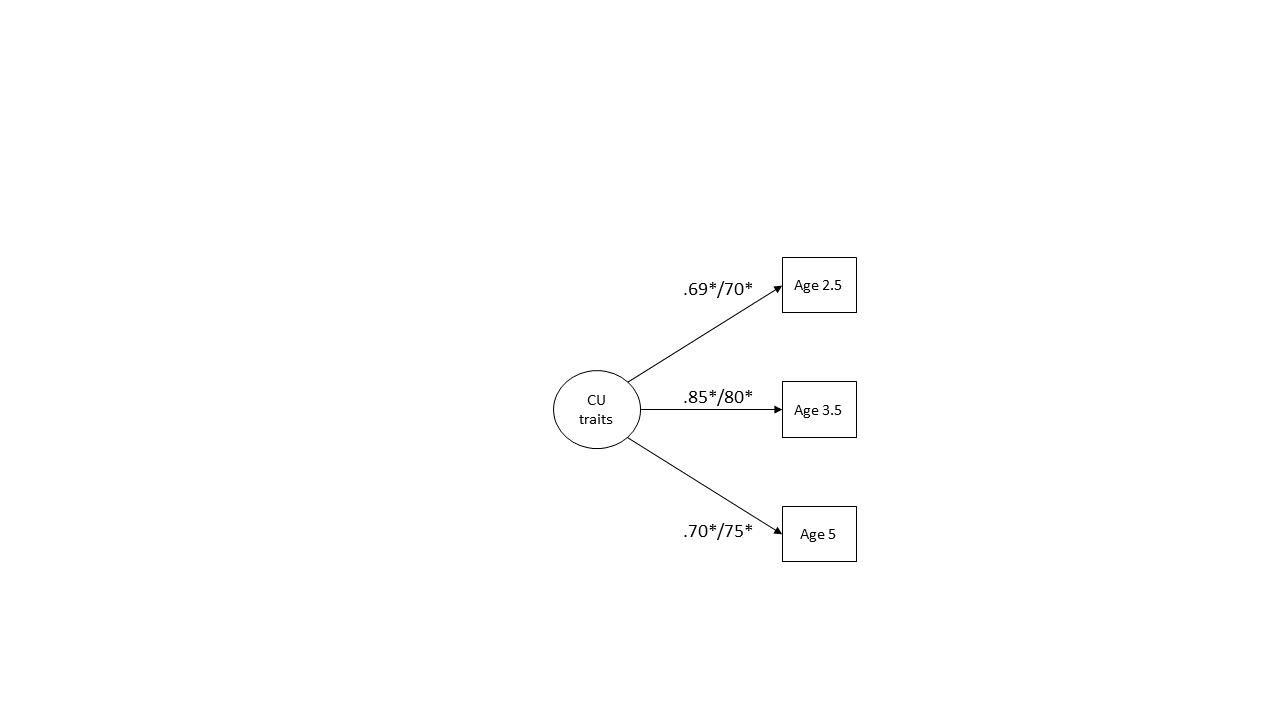


Figure B1: Standardised factor loadings for the age 2.5, 3.5 and 5 years CU traits scores, for boys/girls separately. *p<.001.

**APPENDIX C: Bivariate associations between the key study variables**

Table C1: Bivariate associations between key study variables (total sample)

|  | CU traits | Anxious-depressed | Maternal cortisol | Child sex | Maternal depression | Maternal CU traits |
| --- | --- | --- | --- | --- | --- | --- |
| CU traits latent variable |  | **.29***** | **-.15*** | -.08 | **.20**** | **.24***** |
| Anxious-depressed latent variable |  |  | -.03 | -.02 | **.31***** | **.20**** |
| Maternal cortisol |  |  |  | -.03 | .05 | .01 |
| Child sex |  |  |  |  | .04 | **.24***** |

Note. *p<.05, **p<.01, ***p<.001

Table C2: Bivariate associations between key study variables by child sex (boys on top diagonal and girls on bottom diagonal)

|  | CU traits | Anxious-  depressed | Maternal cortisol | Maternal depression | Maternal CU traits |
| --- | --- | --- | --- | --- | --- |
| CU traits latent variable |  | **.25*** | .06 | .16 | .15 |
| Anxious-depressed latent variable | **.33***** |  | .14 | **.31**** | .05 |
| Maternal cortisol | **-.38***** | -.16 |  | .16 | .02 |
| Maternal depression (age 2.5-5 years latent variable) | **.26**** | **.32***** | -.09 |  | -.08 |
| Maternal CU traits | **.35**  **p=.004** | **.27**  **.005** | -.01  .923 | .13  .200 |  |
